# Cochlear Injury and Adaptive Plasticity of the Auditory Cortex

**DOI:** 10.3389/fnagi.2015.00008

**Published:** 2015-02-05

**Authors:** Anna Rita Fetoni, Diana Troiani, Laura Petrosini, Gaetano Paludetti

**Affiliations:** ^1^Department of Head and Neck Surgery, Medical School, Catholic University of the Sacred Heart, Rome, Italy; ^2^Institute of Human Physiology, Medical School, Catholic University of the Sacred Heart, Rome, Italy; ^3^Department of Psychology, Sapienza University of Rome and IRCCS Santa Lucia Foundation, Rome, Italy

**Keywords:** presbycusis, noise-induced hearing loss, auditory cortex, pyramidal neurons, oxidative stress

## Abstract

Growing evidence suggests that cochlear stressors as noise exposure and aging can induce homeostatic/maladaptive changes in the central auditory system from the brainstem to the cortex. Studies centered on such changes have revealed several mechanisms that operate in the context of sensory disruption after insult (noise trauma, drug-, or age-related injury). The oxidative stress is central to current theories of induced sensory-neural hearing loss and aging, and interventions to attenuate the hearing loss are based on antioxidant agent. The present review addresses the recent literature on the alterations in hair cells and spiral ganglion neurons due to noise-induced oxidative stress in the cochlea, as well on the impact of cochlear damage on the auditory cortex neurons. The emerging image emphasizes that noise-induced deafferentation and upward spread of cochlear damage is associated with the altered dendritic architecture of auditory pyramidal neurons. The cortical modifications may be reversed by treatment with antioxidants counteracting the cochlear redox imbalance. These findings open new therapeutic approaches to treat the functional consequences of the cortical reorganization following cochlear damage.

## Introduction: Challenges for the Investigation of the Relation between Inner Ear Injury and Auditory Cortex Plasticity

**Figure 1:**
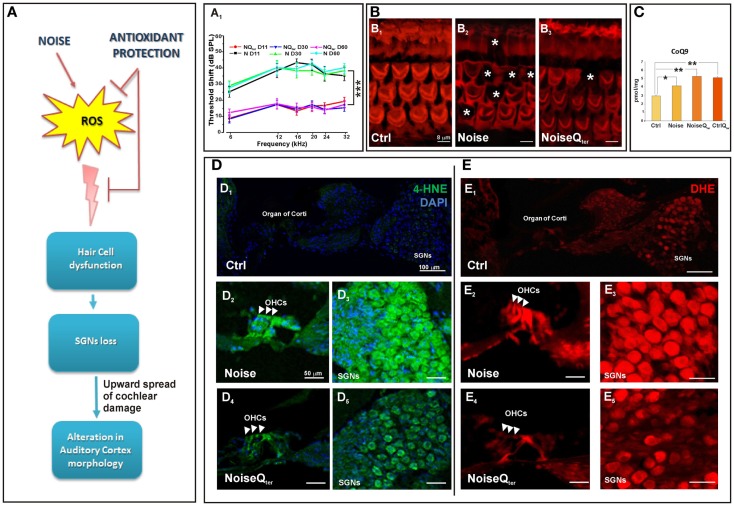
**In the rat, repeated noise exposure causes hearing loss and cochlear oxidative imbalance that is reduced by antioxidant treatment**. The diagram in **(A)** is a schematic representation of the effect of antioxidant supplementation on the upward spread of noise-induced cochlear damage; reactive oxygen species (ROS) over production in cochlear structures induces hair cell dysfunction, spiral ganglion neurons (SGNs) loss and alterations in cortical pyramidal neurons. (A_1_) The hearing loss has been evaluated by ABR threshold shift values (±SEM). Repeated noise exposure (100 dB, 10 kHz, 60 min/day for 10 consecutive days) induces threshold shift of ~40–45 dB for all frequencies tested with a peak between 16 and 24 kHz. NIHL is ameliorated by antioxidant treatment (Q_ter_, 100 mg/kg × 10 days): the threshold shifts is ~10–15 dB at the end of noise sessions. ***p < 0.0001. **(B)** The quantitative assessment of HC survival has been determined by Rhodamine–Phalloidin (Rh–Ph) staining of HC apical pole 60 days after noise exposure. In control, typical distribution in three rows of OHCs and one row of inner hair cells (IHCs) is shown [indicated by asterisks in (B_1_)], in noise exposed animals HC loss is observed mainly in the middle and basal turn [indicated by asterisks in (B_2_)]. The amount of HC disappearance is significantly decreased by antioxidant treatment (B_3_). **(C)** In order to demonstrate that the CoQ analog is protective against oxidative stress in the cochlea, the quantification of quinone levels (CoQ_9_) has been performed by HPLC analysis at the end of Q_ter_ treatment. Interestingly, rats treated with Q_ter_ show higher quinone levels than in Ctrl and noise groups. The cochlear oxidative damage after noise exposure at day 11 has been detected using superoxide **(D)** and lipid peroxidation **(E)** markers. **(D)** Noise-induced superoxide production in the OHCs [indicated by arrow-heads in (D_2_,D_4_)] and SGNs (D_3_,D_5_) is reduced by Q_ter_ treatment. Similarly, Q_ter_ treatment significantly decreases the expression of 4-HNE mainly in OHCs [indicated by arrow-heads in (E_2_,E_4_)] and SGNs (E_3_,E_5_). Data are taken from Fetoni et al. (2013).

Sensory-neural hearing loss is a disorder surprisingly frequent in the general population (Nelson et al., 2005) affecting severely the quality of life as reported by several assessments (Seidman and Standring, 2010). Hearing loss research provided evidence on two major causal insults, aging and noise trauma, and on a common predominant mechanism of damage affecting the organ of corti: the redox status imbalance. Mitochondrial production of reactive oxygen species (ROS) is indeed central to the free radical theory of aging (Lenaz, 2012; Orr et al., 2013). This theory has been implicated in the pathogenesis of virtually all age-associated diseases as well as in noise-induced hearing loss (NIHL), the second most common sensory-neural hearing deficit after age-related hearing loss (presbycusis) (Van Eyken et al., 2007; Someya et al., 2009; Fetoni et al., 2011). In both hearing pathologies, the increase of hearing threshold of about 40–50 dB affects predominantly the high-frequency region and is frequently associated to distressful and debilitating phantom sounds (Heffner and Harrington, 2002; Eggermont and Roberts, 2004; Weisz et al., 2006; Eggermont, 2008; Roberts et al., 2010). The current state of presbycusis and NIHL research suggests that sensory disruption due to damage of the organ of corti may trigger central mechanisms of homeostatic/maladaptive plasticity (Rauschecker, 1999; Syka, 2002; Caspary et al., 2008; Wang et al., 2011; Yang et al., 2011). Consistent with theories of homeostatic plasticity many studies have reported changes in excitatory, inhibitory, and neuromodulatory networks along the central auditory pathway (Liberman and Kiang, 1978; Abbott et al., 1999; Milbrandt et al., 2000; Salvi et al., 2000; Richardson et al., 2012; Engineer et al., 2013). Indeed, research focused selectively either on the analysis of cochlear damage within the organ of corti and its mechanisms or the functional adaptive changes of central and cortical networks. Despite the plethora of data achieved in recent years, a cohesive physiological framework underlying presbycusis and NIHL generation remains elusive inasmuch the relation between cochlear injury and cortical plasticity has been addressed only marginally. To this end, the current review will examine the convergence of factors related to auditory insults from a bottom-up perspective, coupling the acoustically- or aging-induced functional changes at peripheral level [e.g., hearing receptor and spiral ganglion neuron (SGN) function] with the central changes at the level of the pyramidal neurons in the auditory cortices. To gain insights into the relationship between cochlear damage and cortical rearrangement, this review will first address damage-induced ROS imbalance in the cochlea and the effect of antioxidant supplementation, and then the adaptive/maladaptive cortical rearrangement (diagram in Figure 1A).

## Oxidative Stress and Redox Balance in the Hair Cells: The Antioxidant Protection

The loss of hair cells (HCs) induced by acoustic overexposure manifests as extensive outer hair cell (OHC) death, mainly the basally located OHCs, and frequency-delimited loss of inner hair cells (IHCs) scaling with the trauma severity (Spongr et al., 1992). This susceptibility to trauma appears to be conserved in certain models of ototoxicity, such as the exposure to aminoglycoside antibiotics (Forge, 1985), or to platinum-derived cancer treatment drugs (Yorgason et al., 2006). Interestingly, there is an equivalency between loss developed following noise trauma and the loss acquired during aging, as in sensory presbycusis (Schuknecht, 1964; Schuknecht and Gacek, 1993; Ohlemiller, 2004). Ultimately, in models of sensory presbycusis and NIHL, the cochlear injury seems to converge upon auditory neuropathy (Stamataki et al., 2006; Sergeyenko et al., 2013; Gold and Bajo, 2014) and a ROS-dependent mechanism of damage (Henderson et al., 2006; Yamasoba et al., 2013).

**Figure 2:**
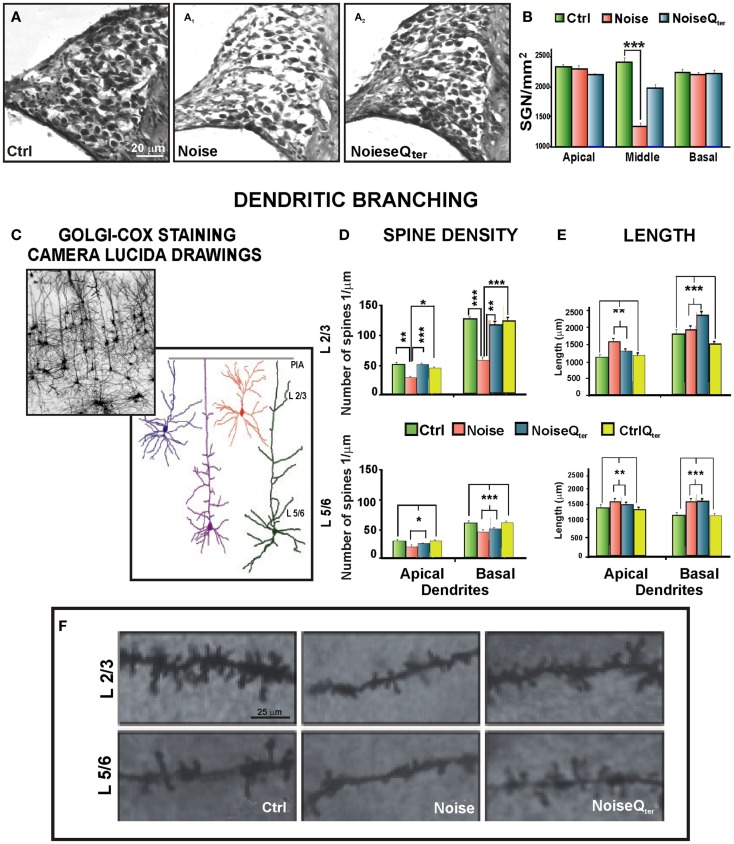
**Cortical morphological modifications induced by noise-induced cochlear damage and peripheral deafferentation are ameliorated by antioxidant treatment**. **(A)** Repeated noise exposure induces SGN degeneration, soma appear smaller, their density is reduced, and fibers are thinner (A_1_) compared with controls **(A)**. Q_ter_ administration preserves SGNs and fibers (A_2_). **(B)** The graph shows SGN viability presented as number of cells per square millimeters, ***p < 0.0001. **(C–F)** Auditory cortex pyramidal neurons belonging to L 2/3 and L 5/6 have been analyzed using Golgi–Cox technique from tissue collected at day 60 after noise exposure. **(C)** Golgi–Cox staining and Camera Lucida drawings of representative pyramidal neurons belonging to L 2/3 and L 5/6 of auditory cortices. **(D–E)** Histograms show the effects of noise exposure and antioxidant treatment (Q_ter_) on dendritic spine density and length of L 2/3 (above) and L 5/6 (below) pyramidal neurons. Vertical bars indicate SEM, *p < 0.05, **p < 0.001, ***p < 0.0001. **(D)** The acoustic trauma significantly decreases spine density in the apical and basal dendrites of both cortical layers. Q_ter_ treatment rescues control values of spine density for both apical and basal dendrites in L 2/3 but not in L 5/6. **(E)** In both layers, the acoustic trauma significantly increases neuronal length both in apical and basal dendrites. Q_ter_ treatment does not modify the dendritic length enhanced by the acoustic trauma in the apical and basal arborizations of L 2/3 and L 5/6 pyramidal neurons. **(F)** Photomicrographs visualize the spines of apical dendritic segments of pyramidal neurons belonging to L 2/3 and L 5/6 of the auditory cortex. Data are adapted from Fetoni et al. (2013).

Reactive oxygen species are formed as byproducts of mitochondrial respiration and examples of oxidizing reactive species are the superoxide anion radical (O_2_), the hydroxyl radical (OH), and hydrogen peroxide (H_2_O_2_) (Bast and Haenen, 2013). Most research on the role of ROS in aging and NIHL has focused on two areas: defining the sites and mechanisms of ROS production and the resulting damage, and developing broad-acting antioxidants to decrease the damage caused by ROS (Someya et al., 2009; Orr et al., 2013). Considerable progress has been made in defining sites of production within the mitochondria and it is generally accepted that complex I and complex III have high capacities for production of superoxide/H_2_O_2_ and they are the sites most relevant to disease (Brand et al., 2013). Under basic metabolic conditions the intrinsic mitochondrial and cytosolic antioxidant machinery maintains redox homeostasis, the steady state between oxidative and reductive forces. However, if ROS are being produced in excess they create oxidative stress that affects various organelles and pathways in the cell, leading to apoptosis, or other forms of cell death, damaging mitochondria themselves and energy metabolism (Finkel, 2012; Böttger and Schacht, 2013). Our data on NIHL and ototoxicity models provide evidence on oxidative stress in the cochlea. Namely, enhanced superoxide production and lipid peroxidation in HCs and SGNs demonstrate the oxidative status after noise exposure and cisplatin-induced ototoxicity (Fetoni et al., 2013, 2014). Among the biomarkers of lipid peroxidation, 4-hydroxy-2-nonenal (4-HNE) is one of the more sensitive and widely used *in vitro* and *in vivo* experimental models (Fetoni et al., 2010, 2013). A strong immunoreactivity for 4-HNE is detected in almost all OHCs in the damaged area in the first 24 h after the acoustic trauma in guinea pigs (Maulucci et al., 2014) and after cisplatin administration in rodents (Fetoni et al., 2014). Interestingly, an increasing level of free radical-induced lipid peroxidation is revealed in OHCs and SGNs in the first 3 days after exogenous insults; peroxidation then decreases in the following 7 days indicating that an early “window” for a successful therapeutic approach against exogenous factors occurs (Fetoni et al., 2010, 2013, 2014). During this period, several endogenous antioxidant pathways, which can be potentiated by exogenous supplementation, are activated to prevent the onset of HC damage. Vascular endothelial growth factor (VEGF), once regarded as an angiogenic factor implicated in antioxidant defense, is up-regulated at 1 and 7 days following intense noise exposure in the organ of corti. VEGF up-regulation can be temporally and spatially correlated to spontaneous recovery of auditory function that occurs in the first 7 post-damage days (Picciotti et al., 2006; Fetoni et al., 2009a). VEGF expression is also significantly reduced in aged mice (Picciotti et al., 2004). These findings suggest a possible interdependent relationship between aging and acoustic trauma on one hand, and oxidative stress mechanisms on the other hand, with potentially important therapeutic implications. Among the many intracellular pathways involved in the adaptive stress response, a relevant role is played by the inducible isoform of heme oxygenase (HO-1), the microsomal enzyme deputed to heme catabolism having antioxidant properties capable of scavenging peroxyl radicals and inhibiting lipid peroxidation (Barone et al., 2009). Several strategies to ameliorate redox status balance have been focused on antioxidant supplementation and there has been extensive research into the discovery of natural and newly designed antioxidants (Le Prell et al., 2007, 2011; Fetoni et al., 2010, 2014). Remarkably in the guinea-pig cochleae, the neuroprotective effect of the antioxidant Ferulic acid, when given 1 day before and for 3 days after noise exposure, is functionally related not only to its scavenging ability but also to the up-regulation of HO-1. These results fit the idea that antioxidants achieve their best cytoprotective capacity if given before and soon after the stressor. Also, in the model of cisplatin-induced oxidative stress HO-1 level is enhanced as an early endogenous, although insufficient, antioxidant response and this pathway is potentiated by the administration of the dietary antioxidant curcumin (Fetoni et al., 2014). Although the issue on the different mechanisms of cochlear oxidative stress/ROS generation in NIHL, ototoxicity and sensory presbycusis is not resolved, common to these hearing pathologies is mitochondrial dysfunction (Böttger and Schacht, 2013). The antioxidant ability to donate electrons of coenzyme Q_10_ (CoQ_10_) in targeting mitochondrial dysfunction can be considered a promising approach inasmuch CoQ_10_ functions as an electron carrier from the protein complex I and II to complex III (Crane, 2001; Lenaz et al., 2007). As energy carrier, the CoQ_10_ factor continuously goes through oxidation–reduction cycle. In its reduced form, the CoQ_10_ holds electrons rather loosely, so CoQ_10_ will quite easily give up one or both electrons and, thus, act as antioxidant. CoQ_10_ inhibits lipid peroxidation by preventing the production of lipid peroxyl radicals, reduces the initial perferryl radical, which prevents propagation of lipid peroxidation, protects not only lipids but also proteins from oxidation. In addition, the reduced form of CoQ_10_ effectively regenerates vitamin E from the α-tocopheroxyl radical (Sohal and Forster, 2007). Considering that the efficacy of antioxidants is best tested in terms of their ability to maintain homeostasis CoQ_10_ analogs have been tested in NIHL. The synthetic analog of CoQ_10_, idebenone, significantly prevents NIHL when administered in the peritraumatic period decreasing the apoptotic cascade activation and then avoiding HC loss (Sergi et al., 2006; Fetoni et al., 2008). Its efficacy seems to depend on the ability to intercept free radicals in both aqueous phases and lipid–water interfaces. On this basis, the protective role of CoQ_10_ against NIHL has been analyzed by comparing the efficacy of the native lipophilic CoQ_10_ molecule with that of a multi-composite formulation of CoQ_10_ with high water solubility and oral bioavailability, CoQ_10_ Terclatrate (Q_ter_). The water soluble molecule is more effective as compared to the native CoQ_10_ in decreasing apoptosis as shown by the reduced expression of active caspase 3 and thus in improving hearing. The obtained results confirm that solubility of Q_ter_ improves the ability of CoQ_10_ in preventing oxidative injuries that result from mitochondrial dysfunction (Fetoni et al., 2009b, 2012, 2013). In fact, the systemic administration of Q_ter_ decreases superoxide production and 4-HNE expression in HCs and SGNs (Figure 1). Interestingly, reduced oxidative stress is consistent with the increased levels of the endogenous quinones (i.e., CoQ_9_, the major form expressed in rats) after the administration of Q_ter_ indicating that the exogenous quinone can exert a protective effect on animal tissues. In fact, in the NoiseQ_ter_ group, CoQ_9_ levels decrease at the end of treatment compared with the control Q_ter_ group, demonstrating that the exogenous quinone is used as scavenger during noise exposure to reduce the oxidative imbalance. This scavenging would thus prevent the functional and morphological cochlear damage (Figures 1 and 2A,B), the upward spread of the cochlear damage and the deafferentation consequences in the auditory cortex (Figure 1A).

## Insult-Mediated Adaptive/Maladaptive Plasticity in the Auditory Cortex

Noise-induced hearing loss, ototoxicity, or age-induced damage to the peripheral hearing organ causes primarily alteration of the firing rates in the auditory nerve (Kraus et al., 2011), and compensatory changes at various levels of the central auditory pathway (Jin et al., 2005; Jin and Godfrey, 2006; Meidinger et al., 2006; Wang et al., 2006; Kraus et al., 2009; Kujawa and Liberman, 2009). The consequences of acoustic trauma have been investigated mainly through electrophysiological and neurochemical analyses, whereas morphological data in the central acoustic system are still scant (Bose et al., 2010; Gröschel et al., 2010). Nevertheless, following noise-induced acoustic trauma, decreased spine density paralleled by an increased dendritic length has been observed in the pyramidal neurons of auditory cortical areas (Figure 2) (Fetoni et al., 2013). Namely, pyramidal neurons belonging to layer II–III (L 2/3) and V–VI (L 5/6) of auditory cortices have been analyzed by using the Golgi–Cox technique from tissue collected two months after noise injury (Figure 2C). In both cortical layers and both apical and basal dendrites, the acoustic trauma significantly decreased spine density (Figure 2D) and increased dendritic length (Figure 2E). The distance from the soma of maximal spine concentration remained unaltered in the arborizations of L 2/3 while it was distally shifted in the apical and basal dendrites of L 5/6 reducing the efficacy of synapses on neuronal output (Fetoni et al., 2013). In the absence of dendrite shrinkage, spine loss may be explained by excessive synaptic pruning attributable to enhanced synaptic competition. Hence, the spine loss that follows deafferentation may be caused by activity-dependent remodeling of neuronal connectivity and it could be a “trophic” response, whereby a diminished input cannot sustain a large number of excitatory connections. Alternatively, the deafferented cortical neuron could compensate for the reduced afferent drive by sensing global levels of activity and operating a homeostatic synaptic scaling (Turrigiano, 2008, 2012; Whitt et al., 2014). If so, the decrease in spine number could result in an up-regulated excitatory signaling and preserve relative synaptic efficacy. Literature on homeostatic plasticity (Caspary et al., 2008; Richardson et al., 2012; Gold and Bajo, 2014) describes how in response to changes in chronic neuronal activity, i.e., deafferentation, neural systems undergo compensatory changes in synaptic activity to stay within a relatively narrow operating range of the original neuronal activity (Turrigiano, 1999, 2007; Rich and Wenner, 2007). A number of plasticity studies have focused on the potential significance of the balance between excitation and inhibition to explain the adaptive and maladaptive homeostatic plasticity of cortical tonotopic map reorganization and tinnitus, respectively (Eggermont and Roberts, 2004; Roberts et al., 2010; Pienkowski and Eggermont, 2011; Wang et al., 2011). The cellular compensatory mechanisms involve the regulation of inhibitory and excitatory neurotransmission, since changes in one system produce reactive changes in the other one (Turrigiano, 2012). In response to increased neuronal activity, inhibitory and excitatory synaptic strengths are multiplicatively scaled up and down, respectively (Peng et al., 2010; Rannals and Kapur, 2011), to restore neuronal firing rate to normal levels. Indeed, dendrites and their spines are the main neuronal targets of plasticity (Feldman, 2012; Fortin et al., 2012; De Bartolo et al., 2014; Sala and Segal, 2014). Dendritic arbors and spines are then highly dynamic structures branching and retracting in response to the information they receive, so that dendritic length and spine number are related to the degree of connectivity and the complexity of information processing (McAllister, 2000). They provide the morphological substrate for lesion-induced and context-dependent plastic events (Kulkarni and Firestein, 2012).

Interestingly, systemic treatment with the antioxidant CoQ_10_ analog Q_ter_ in the rat NIHL model not only reduced the oxidative stress and cochlear damage but also prevented the alteration of the pyramidal dendritic pattern of the auditory cortex in a layer-selective mode (Figures 2D–F). Namely, the spine densities for both apical and basal dendrites were rescued to control values (Figure 2D) without modifying its distance from soma in L 2/3, but not in L 5/6 (Fetoni et al., 2013). However, the antioxidant treatment did not modify the dendritic length enhanced by the acoustic trauma in the apical and basal arborizations of L 2/3 and 5/6 pyramidal neurons (Figure 2E). As the other sensory cortices, the auditory cortex shows dense and well-developed L2/3, mainly involved in cortico-cortical circuits, and relatively sparse and reduced L5/6 (Linden and Schreiner, 2003; Paxinos and Watson, 2007). Thus, the neuronal rearrangement of the auditory cortex appears to engage mainly the cortico-cortical circuits and L2/3 homeostatic plastic changes are the substrate for cortical plasticity, as reported in other sensory cortices (Kotak et al., 2005; De Bartolo et al., 2009; Gelfo et al., 2009; Whitt et al., 2014). Overall, various forms of plasticity, including synaptic scaling, plasticity of intrinsic excitability, and changes in sensory-evoked inhibition and excitation–inhibition ratio, cooperate to modify the function of cortical circuits (Li et al., 2014; Whitt et al., 2014). This rich repertoire of synapse regulation and plasticity enables cortical circuits to respond with the greatest flexibility to changes in sensory input. On one hand, the several forms of homeostatic plasticity operating on different temporal and spatial scales may guarantee the apt compensatory responses to a wide range of sensory perturbations. Interestingly, in both juvenile and adult mammals, hearing loss restricted to a part of the audible frequency range can lead to a reorganization of cortical tonotopic maps (Pienkowski and Eggermont, 2011). Thus, the cortical modifications after NIHL, as illustrated in Figure 2, could be the structural basis of such a functional phenomenon for which within a few weeks from the onset of severe but restricted hearing loss, the cortical region related to the dysfunctional cochlear part becomes tuned to the sound frequencies, which stimulate the adjacent non-damaged part(s) (Eggermont and Komiya, 2000; Noreña and Eggermont, 2005). On the other hand, maladaptive cortical plasticity or impaired synaptic plasticity might contribute to the excess of plasticity as reported in focal and generalized form of dystonia (Quartarone and Pisani, 2011). It can be speculated that a deficit of synaptic “down-scaling” along with a deficient inhibition may underlie the excess of plasticity in tinnitus and the increased plasticity in the auditory cortex and/or multiple levels of the central auditory neuraxis can become maladaptive, giving rise to abnormal sensory patterns.

## Conclusion

Mitochondrial production of ROS is implicated in the pathogenesis of virtually all age-associated diseases as well as in NIHL. As shown in the acoustic trauma model, noise exposure induces oxidative stress damage in the sensory epithelium of the organ of corti and degeneration of SGNs. The upward spread of cochlear oxidative damage appears to cause plastic rearrangement in the pyramidal layers (L 2/3 and L 5/6) of the auditory cortex. Antioxidants, such as Q_ter_, Ferulic acid, and Idebenone, reduce the morphological and functional cochlear damage. The decrease of the peripheral oxidative imbalance reverses the upward spread of the cochlear damage and the deafferentation consequences in the auditory cortex, specifically in the highly plastic L 2/3. The present data demonstrate the capability of the auditory cortex to remodel its features in consequence of antioxidant therapy.

## Conflict of Interest Statement

The authors declare that the research was conducted in the absence of any commercial or financial relationships that could be construed as a potential conflict of interest.
